# Supplementary material for: Extended spectrum beta lactamase producing bacteria among outpatients with ear infection at FelegeHiwot Referral Hospital, North West Ethiopia
Source: PLoS One. 2020 Sep 11;15(9):e0238891. doi: 10.1371/journal.pone.0238891 (PMC7485783; doi:10.1371/journal.pone.0238891)
Supplement: S1 File — (DOCX) [file pone.0238891.s001.docx]

**Questionnaire for Investigation of Extended spectrum beta lactamases producing bacteria among patients with ear infection at Felege Hiwot Referral Hospital , North West Ethiopia.**

| List of Questions (English version) |  |  |  |
| --- | --- | --- | --- |
| 1. Code number:--------------------- |  |  |  |
| 1. Date of sample collection-------- |  |  |  |
| 1. Age of patient (in years)-------- |  |  |  |
| 1. Sex of patient | - Male | - Female |  |
| 1. Residence | - Urban | - Rural |  |
| 1. Type of ear infection | - Acute otitis media | - Chronic suppurative otitis media | - Otitis Externa |
| 1. Ear involved in infection | - Right | - Left | - Both |
| 1. Hearing status the patient | - Well | - Decreased |  |
| 1. Ear discharging type | - White | - Yellow Green | - Bloody |
| 1. Does the patient have bottle feeding history? | - Yes | - No |  |
| 1. Does the patient have chronic diseases | - Yes | - No |  |
| 1. Antibiotic use without physician prescription | - . Yes | - No |  |
| 1. Do you have upper respiratory infection? |  |  |  |
| 1. Previous Hospital visit and treatment | - Yes | - No |  |

**Amharic Version (Local language)**

እስከ 3ኛ ደረጃ ያሉትን የ ሴፋሎሰፖሪን ዝርያ ፀረ ባክቴሪያን የተላመደ የባክቴሪያ ዝርያዎችን በ ፈለገህይዎት ሪፈራል ሆስፒታል በሚታከሙ የጀሮ ሕመምተኞ ላይ ከጀሮ በሚወሰድ ናሙና ውስጥ መኖራቸውን መለየት፤ በምንያህል መጠን እንዳለ ማወቅ፤ በምን መድሀኒት ሊድኑ እንደሚችሉ ለማወቅ የተዘጋጀ መጠይቅ ነዉ፡፡

**የ መጠይቅ ዝርዝር**

| 1. የኮድ ቁጥር**: ------** |  |  |
| --- | --- | --- |
| 1. ናሙናዉ የተወሰደበት ቀን**፡**  **------------------** | |  |
| 1. የታካሚዉ እዴሜ (በዓመት)**፡ --------------** | | |
| 1. ጾታ | - ወንድ | - ሴት |
| 1. የመኖሪያ አድራሻ | - ከተማ | - ገጠር |
| 1. የጀሮ ሕመም አይነት | - የቅርብ ጊዜ | - የረጅም ጊዜ ፈሳሽያለዉ - የዉጨኛዉ ክፍል |
| 1. የታማሚዉ የጀሮ ክፍል | - የቀኝ ጀሮ | - የግራ ጀሮ - ሁለቱም |
| 1. ከጀሮ የሚወጣዉ የጀሮ ፈሳሽ አይነት | - ነጭ - ቢጫ | - ደም የቀላቀለበት - አረንጓዴ |
| 1. የጀሮ የመስማት አቅም | - ምንም አይነት ችግር የሌለበት | - የመስማትአቅምየቀነሰ |
| 1. በህፃንነትዎ ጡጦ ተጠቅመዉ ያዉቃሉ ? | - አዎ | - የለም |
| 1. ጽኑ ህመም ታመዉ ያዉቃሉ? | - አዎ | - የለም |
| 1. ከዚህ በፊት ሆስፒታል ታክመዉና መዳኒት ወስደዉ ያዉቃሉ | - አዎ | - የለም |
| 1. የ ላይኛው የ መተንፈሻ ክፍል ህመም አሎት | - አዎ | - የለም |
| 1. ያለሀኪም ትዕዛዝ መድሀኒት ወስደዉ ያዉቃሉ | - አዎ | - የለም |
