# Supplementary material for: Extended spectrum beta lactamase producing bacteria among outpatients with ear infection at FelegeHiwot Referral Hospital, North West Ethiopia
Source: PLoS One. 2020 Sep 11;15(9):e0238891. doi: 10.1371/journal.pone.0238891 (PMC7485783; doi:10.1371/journal.pone.0238891)
Supplement: Attachment — Submitted filename: Response to Reviewers.docx [file pone.0238891.s002.docx]

**Point by point responses to reviewers comment**

PONE-D-20-18371

**Title of the manuscript:** Extended spectrum beta lactamase producing bacteria profiles among outpatients with ear infection at Felege Hiwot Referral Hospital, North West Ethiopia
PLOS ONE
**Dear Editor,**

We have found the editors’ remarks and comments of the reviewers valuable. We would like to thank you for considering our manuscript for publication to your journal. We have revised the manuscript based on your comment. Regarding the remarks from the editor, we have thoroughly revised the manuscript to improve its readability and all the other comments given by reviewers are incorporated in the revised version of the manuscript. The manuscript is thoroughly copyedited for language usage, spelling and grammar by our colleague. All grammatical and typographical errors are corrected and the changes made are highlighted. The manuscript was also checked and revised for compliance with PLOS ONE's style requirements.

**Point –by –point responses to Editors comment**

Regarding guideline used for antimicrobial susceptibility, please indicate the guideline used- CLSI or EUCAST

**Response:** Well taken and only CLSI guideline was used. This is corrected in the revised manuscript.

Additional Editor Comments:

Please revise the manuscript addressing all points raised by the reviewers.

**Response**: Well taken and all points raised by reviewers are addressed in the revised manuscript

 Journal Requirements:

When submitting your revision, we need you to address these additional requirements.

1. Please ensure that your manuscript meets PLOS ONE's style requirements, including those for file naming. The PLOS ONE style templates can be found at

https://journals.plos.org/plosone/s/file?id=wjVg/PLOSOne_formatting_sample_main_body.pdf and

https://journals.plos.org/plosone/s/file?id=ba62/PLOSOne_formatting_sample_title_authors_affiliations.pdf

**Response:** Well taken. We have strictly followed the pLOS ONE style requirements.

2. We suggest you thoroughly copyedit your manuscript for language usage, spelling, and grammar. If you do not know anyone who can help you do this, you may wish to consider employing a professional scientific editing service.  

**Response:** Well taken and the manuscript is thoroughly copy edited for the language usage, spelling and grammars by the colleague Professor Mulugeta Kibret.

3. Please provide a copy of the questionnaire used in your study as a supporting information file.

**Response:** The soft copy of the questionnaire used in both English and local language is uploaded as a supporting information file.

4. Please amend your list of authors on the manuscript to ensure that each author is linked to an affiliation. Authors’ affiliations should reflect the institution where the work was done (if authors moved subsequently, you can also list the new affiliation stating “current affiliation:….” as necessary).

**Response:** Well taken and corrected in the revised manuscript

5. We note you have included a table to which you do not refer in the text of your manuscript. Please ensure that you refer to Table 4 in your text; if accepted, production will need this reference to link the reader to the Table.

[Note: HTML markup is below. Please do not edit.]

**Response:** Well taken and Table 4 is referred in the text of the revised manuscript

Reviewers' comments:

**Point by point responses to reviewers comment**

Reviewer #1:

1. Introduction can be made more concise

**Response**: We have revised the introduction but the size is still not changed.

2. Line 3- sentence looks incomplete

**Response:** Well taken and the incomplete sentence completed in the revised manuscript

3. Line 41- 43- probably authors mean factors associated with ear infections

**Response:** Well taken and the phrase ESBL production was substituted by ear infection in the revised manuscript.

4. Line105-106-please check the sentence

**Response:** Checked and minor revisions made.

5. Line113- 114- one of the references either CLSi or Eucast can be used instead of both

**Response:** Well taken. Only CLSI guideline referred in the revised manuscript. This is corrected both in the manuscript and list of references.

6. Line 117 - 118- different generation cephalosporin could have been put for susceptibility.

**Response**: Only third generation cephalosporins were used in the investigation and this specified in the revised manuscript

7. Line 123- 130- Please put reference

**Response**: Well taken and a reference number 27 is cited in the revised manuscript

8. Line 167- please reframe sentence for better understanding

**Response:** Well taken and corrected in the revised manuscript

9.Line 287- please check the sentence in in view of the fact that esbl producers are resistant to monobactams.

Response: Well taken and checked in the revised manuscript
